# High-Level Expression of Pro-Form Lipase from *Rhizopus oryzae* in *Pichia pastoris* and Its Purification and Characterization

**DOI:** 10.3390/ijms15010203

**Published:** 2013-12-24

**Authors:** Jian-Rong Wang, Yang-Yuan Li, Shu-De Xu, Peng Li, Jing-Shan Liu, Dan-Ni Liu

**Affiliations:** 1 Guangdong VTR Bio-Tech Co., Ltd., Zhuhai 519060, Guangdong, China; E-Mails: believe1234@126.com (J.-R.W.); liyangyuanvtr@163.com (Y.-Y.L.); xushude0106@163.com (S.-D.X.); lengfeng-lp@126.com (P.L.); LiuJsh@126.com (J.-S.L.); 2 Guangdong Feed Additive Research and Development Center, Zhuhai 519060, Guangdong, China

**Keywords:** *Rhizopus oryzae*, lipase, *Pichia pastoris*, expression

## Abstract

A gene encoding *Rhizopus oryzae* lipase containing prosequence (ProROL) was cloned into the pPICZαA and electrotransformed into the *Pichia pastoris* X-33 strain. The lipase was functionally expressed and secreted in *Pichia pastoris* with a molecular weight of 35 kDa. The maximum lipase activity of recombinant lipase (rProROL) was 21,000 U/mL, which was obtained in a fed-batch cultivation after 168 h induction with methanol in a 50-L bioreactor. After fermentation, the supernatant was concentrated by ultrafiltration with a 10 kDa cut off membrane and purified with ion exchange chromatography using SP Sepharose Fast Flow chromatography. The optimum pH and temperature of the rProROL were pH 9.0 and 40 °C, respectively. The lipase was stable from pH 4.0 to 9.0 and from 25 to 55 °C. The enzyme activity was enhanced by Ca^2+^ and inhibited by Hg^2+^ and Ag^+^. The lipase showed high activity toward triglyceride-Tripalmitin (C16:0) and triglyceride-Trilaurin (C12:0).

## 1. Introduction

Lipases (EC 3.1.1.3) are a class of hydrolases that catalyze a variety of reactions, such as the hydrolysis of fatty acid ester, trans-esterification, and ester synthesis at the interface between the insoluble substrate and water [1]. Microbial lipases are currently receiving much attention because of their biotechnological potential, such as broad substrate specificity, high yield and low cost production and so on. Therefore, they have been widely used in industrial applications, such as biodiesel production, organic synthesis, food, pharmaceutical, and detergents [2,3].

It has been known for many years that the fungus *Rhizopus oryzae* produces extracellular lipase. Lipase from *Rhizopus oryzae* has wide applications in industries, such as esterification of docosahexaenoic acid (DHA) and biodiesel fuel production [4,5]. The native structure of *Rhizopus oryzae* Lipase (ROL) comprises a signal sequence of 26 amino acids, a prosequence of 97 amino acids and a mature lipase region of 269 amino acids (mROL) [6–8]. The prosequences are usually located between a signal peptide and a functional region of the protein. They often function as intramolecular chaperones that help folding of the functional region of the protein. Yang *et al.* reported that the prosequence of ROL is involved in the correct folding and efficient secretion of mROL [9]. Although prosequence of ROL aids folding of mROL, to our knowledge, it is still unknown how the prosequence contributes to folding, post-translational processing and maturation of mROL in *Rhizopus oryzae*.

Until now, ProROL (ROL without a signal sequence of 26 amino acids) has already been expressed in *Escherichia coli* (*E. coli*) and *Saccharomyces cerevisiae* (*S. cerevisiae*). In *E. coli*, ProROL could be efficiently produced in high yield at high specific activity (166 U/mL) [10]. However, this expression system has the bottleneck that a cell disruption process is necessary, when compared with other expression systems in which the enzyme is secreted in the culture broth. In *S. cerevisiae*, ProROL could be secreted in the culture broth, but the activity of ProROL was very low (extracellular lipase activity reached only 2.88 U/mL at 120 h of cultivation in Yeast Extract Peptone Dextrose (YPD) medium [11].

Compared with the *E. coli* and *S. cerevisiae*, the methylotrophic yeast *Pichia pastoris* (*P. pastoris*) has many advantages as a host for production of recombinant heterologous proteins, such as high cell density, high levels of productivity, ease of genetic manipulation, the ability to perform complex post-translational modifications and very low secretion levels of endogenous proteins [12]. Several lipases have been expressed successfully in *P. pastoris*, such as *Candida albicans*, *Aspergillus tamari*, *Yarrowia lipolytica* and *Aspergillus niger* [13–16]. Until now, there are no reports about high-level expression of ProROL in *P. pastoris* in bioreactor. In this article we describe the high-level expression of the ProROL in *P. pastoris* in a 50-L bioreactor, with the aim of allowing the production of high amounts of the relevant enzyme. The purification and characterization of the recombinant ProROL (rProROL) was also investigated.

## 2. Results and Discussion

### 2.1. Selection of Lipase Producing Clones

One hundred randomly picked zeocin resistant positive clones on the solid selective medium (YPDS containing zeocin) were transferred on YPD-rhodamine B-olive oil medium plates to check lipase expression level. Lipase expression was induced by adding 0.2 mL of methanol onto the lid covering the plates. Ten colonies that had larger halos were selected and cultivated in shaking flask. In shaking flask, the lipase activity increased gradually and reached the highest activity after 168 h of cultivation (supplementary information Figure S1). After 168 h of cultivation under inducing conditions, the lipase activity of the supernatant from different clones varied between 450 and 780 U/mL. A clone showed the highest lipase activity of 780 U/mL in shaking flask culture and this was chosen for all further experiments.

### 2.2. High Cell Density Fermentation for Production of Recombinant Lipase

**Figure 1.**
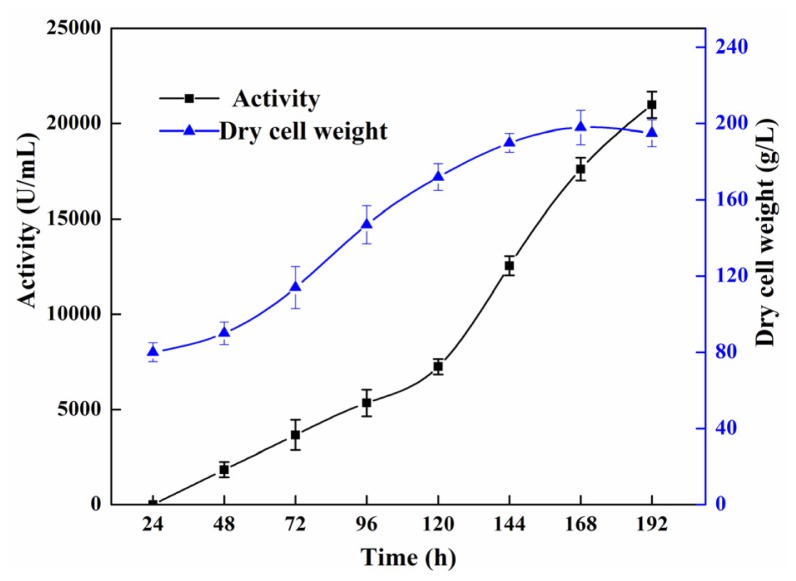
Lipase activity and cell growth in basal salt medium at 30 °C and pH 5.0 during fed-batch fermentation in a 50-L bioreactor. The lipase activity was measured with a pH-stat (Metrohm, Herisau, Switzerland) and using olive oil as substrate at pH 9.0, 40 °C. Dry cell weight was obtained by centrifuging 10 mL samples in a pre-weight centrifuge tube at 8000 *g* for 10 min and washing twice with deionized water, then allowing the pellet to dry at 105 °C to constant weight.

The fed-batch fermentation continued for a period of 8 days. After 24 h batch culture when biomass reached 80 g/L dry cell weight, glycerol was exhausted and the cells were then induced with methanol. Upon methanol induction, the lipase activity in the supernatant reached a maximum of 21,000 U/mL, after 168 h induction with a dry cell weight of 197 g/L (Figure 1). Compared with the shaking flasks procedure, the high cell density fed batch of *P. pastoris* produced a significantly higher level of the recombinant enzyme. Under these conditions, a 27-fold increase in the volumetric lipase activity was obtained when scaling from shaking flasks to bioreactor (780 and 21,000 U/mL, respectively). In *E. coli*, the activity of rProROL could reach 166 U/mL (pNPB assay) in 100 mL Luria-Bertani medium [10]. Compared with the expression of ProROL in *E. coli*, the expression of ProROL in *P. pastoris* has many advantages such as extracellular production of active rProROL, high level production of rProROL. In *S. cerevisiae* the activity of rProROL could reach 2.88 U/mL (pH-stat assay) after 120 h of cultivation in 200 mL YPD medium, which was much lower than that of the rProROL in this study [11]. Until now, several *Rhizopus* lipases have also been successfully expressed in *P. pastoris*. The *R. arrhizus* lipase [6] and *R. chinensis* lipase [17] were functionally expressed and secreted in the *P. pastoris*, the enzyme activity of which reached 315 and 586 U/mL, which were lower than the expression level of the rProROL in this study. However, these values are not fully comparable since different cultivation conditions and activity assays with different substrates and conditions have been used. It is well known that the lipolytic activity test largely depends on a variety of factors, e.g., the type of substrate, the substrate’s interfacial area that is available for the enzyme, as well as the equipment used [17].

### 2.3. Purification of Recombinant Lipase

**Figure 2.**
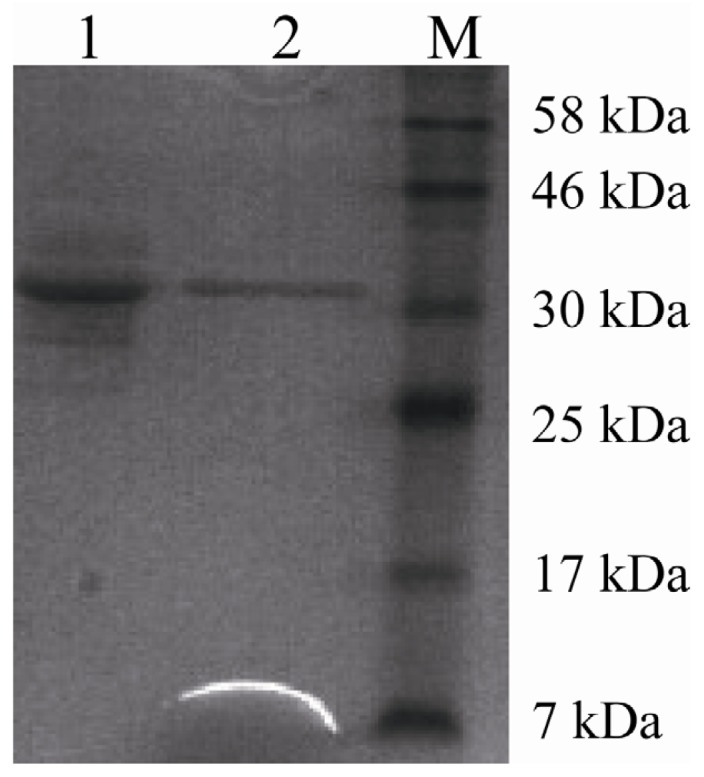
Sodium dodecyl sulfate-polyacrylamide gel electrophoresis (SDS-PAGE) of the expression and purification of rProROL in *P. pastoris* X-33. Lane **M**: molecular weight standards; Lane **1**: 11 μg protein from ultrafiltration; Lane **2**: 7 μg protein from SP Sepharose FF chromatography.

One hundred milliliters of the supernatant was ultrafiltrated through 10 kDa membrane, yielding 20 mL of concentrated lipase solution with a 94% yield and a 1.2-fold increased specific activity of 2267 U/mg. After anion exchange chromatography, a specific lipase activity of 6690 U/mg with a 3.02-fold increased was obtained (Table 1). The purified rProROL showed a single band on sodium dodecyl sulfate-polyacrylamide gel electrophoresis (SDS-PAGE) (Figure 2). The molecular weight of rProROL was 35 kDa, which is similar to the size of the recombinant Pro28ROL derived from *S. cerevisiae* [11].

### 2.4. Biochemical Characteristics of Recombinant Lipase

#### 2.4.1. Effect of pH and Temperature

**Figure 3.**
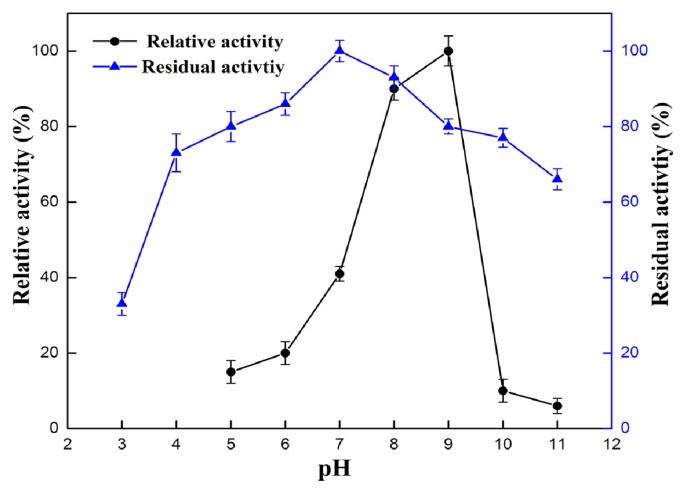
Influence of pH on rProROL activity and stability. The lipase activity was measured with a pH-stat (Metrohm), using olive oil as substrate. Optimal pH was determined by assessing the activity of the purified rProROL at pH 5.0–11.0. The relative activity at different pH values was calculated by setting pH 9.0 as 100%. The pH stability was determined by measuring the residual enzyme activities after incubating purified rProROL at various pH (50 mM acetic acid-sodium acetic acid buffer (pH 3.0–5.0), 50 mM Na_2_HPO_4_-NaH_2_PO_4_ buffer (pH 6.0–7.0), 50 mM Tris-HCl buffer (pH 8.0–9.0), NaHCO_3_ buffer (pH 10.0–11.0) for 24 h at 30 °C. The residual activity was calculated by taking the activity of purified rProROL without buffer treatment as 100%. All measurements were carried out in triplicate.

**Figure 4.**
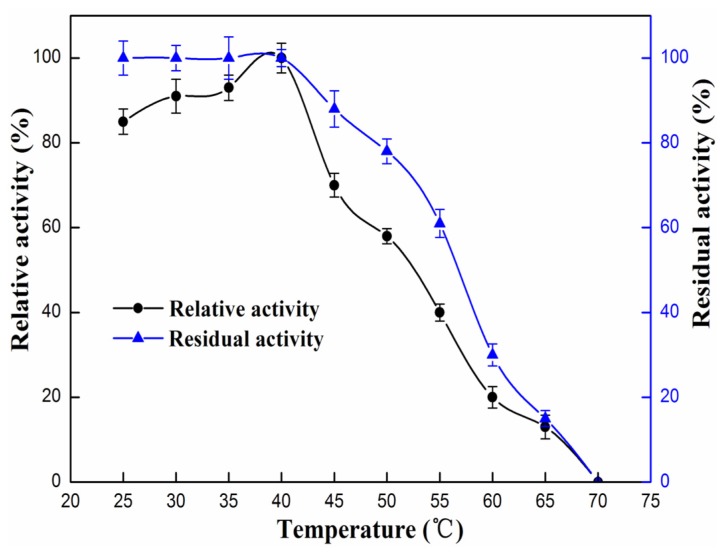
Influence of temperature on rProROL activity and stability. The lipase activity was measured with a pH-stat (Metrohm) and using olive oil as substrate. The optimum temperature of purified rProROL was measured at different temperatures ranging from 25 to 70 °C. The relative activity at different temperatures was calculated by setting 40 °C as 100%. The thermal stability was studied by incubating lipase at various temperatures (25–70 °C) in Tris-HCl buffer (pH 8.0) up to 1 h. The residual enzyme activity was measured at 40 °C with olive oil as substrate and the residual activity was calculated by taking the non-heated lipase activity as 100%. All measurements were carried out in triplicate.

The influence of pH and temperature on the enzyme activity and stability is presented in Figures 3 and 4. As shown in Figure 3, the enzyme showed maximum activity at pH 9, the lipase activity decreased dramatically when pH was above 9 or below 8. Optimum pH for rProROL is similar to that for lipases from *R. chinensis* which showed maximum activity at pH 8.5 and was stable over a narrow pH range of 7.5–8.5 [17]. As shown in Figure 3, the rProROL could maintain more than 75% of maximum activity after incubating at a pH range of 4–9 at 30 °C for 24 h. The pH stability of rProROL is comparable to the lipase from *Ralstonia* which could maintain more than 60% of maximum activity after incubating at a pH range of 5–10 at 4 °C for 24 h [18]. As shown in Figure 4, the recombinant lipase showed an optimum activity at 40 °C and activity dropped sharply above 50 °C with no activity remaining at 70 °C. The optimum temperature of rProROL is similar to that of the prolipase from *R. chinensis* which exhibited maximum activity at 40 °C [17]. Thermostability was examined by incubating rProROL at different temperatures for 1 h, and the residual activity was measured at 40 °C under the conditions mentioned above. The activity of rProROL was almost not affected by a temperature below 40 °C, but it decreased dramatically when the temperature was above 55 °C. rProROL showed 60% residual activity after 1 h incubation at 55 °C. Thermal stability of rProROL was higher than mROL which showed no residual activity remained after 30 min incubation at 55 °C [19].

#### 2.4.2. Effect of Metal Ions on Lipase

Effect of different metal ions on the activity of the lipase is presented in Table 2. The lipase activity was slightly enhanced by Mg^2+^, K^+^ and Na^+^. Metal ion Ca^2+^ increased the activity of rProROL by 11%. The activity enhancement caused by Ca^2+^ and inhibition caused by and Zn^2+^ of rProROL was almost similar to those of *Pseudomonas aeruginosa* LX1 lipase (Ca^2+^ increased *P. aeruginosa* LX1 lipase activity by 21% and Zn^2+^ reduced the lipase activity to 13.8%) and *Galactomyces geotrichum* Y05 lipase (Ca^2+^ increased *G. geotrichum* Y05 lipase activity by 14% and Zn^2+^ reduced the lipase activity to 57%) [20,21]. A possible explanation for this phenomenon is that Ca^2+^ form complexes with ionized fatty acids which facilitated the removal of free fatty acids formed in the reaction at the fat–water interface and changing their stability and behaviors at the interfaces [22]. The activity could maintain more than 80% of the control value in the presence of Li^+^, Co^2+^, Mn^2+^, Cu^2+^. The enzyme activity was strongly inhibited by Hg^2+^ and Ag^+^, suggesting that Hg^2+^ and Ag^+^ were able to alter the enzyme conformation. In agreement with our results, Yan *et al.* [21] and Sharon *et al.* [23] also found Hg^2+^ and Ag^+^ had a strong inhibitory effect on lipase activity. The metal-chelating agent EDTA did not affect the activity of rProROL.

#### 2.4.3. Effect of Detergents on Lipase

For use in laundry detergents, lipase needs to be resistant to surfactants. Non-ionic detergent Triton X-100 increased slightly the enzyme activity to 104%, after 6 h at 25 °C. However, the activity was inhibited by other anionic detergent Tween-20 and Tween-80 which reduced rProROL activity to 76% and 71%, respectively. The activity remained almost stable in the presence of β-mercaptoethanol. Anionic detergent SDS had strong inhibitory effect on lipase activity, which reduced rProROL activity to only 7%. In accordance to our results, Cheng *et al.* [24] also found a great loss of activity in the presence of SDS but an enhanced activity in the presence of TritionX-100.

#### 2.4.4. Effect of Organic Solvents

The stability of ProROL in the presence of various organic solvents is presented in Table 3. In this work, there was little effect of glycerol on rProROL. On the contrary, methanol, isopropanol, chloroform, acetone, ethanol and butanol decreased enzyme activity to 5%–61%, probably because they provoked a rapid protein denaturation. The enzyme stability was also evaluated in the presence of various concentrations of methanol and ethanol (Table 4). After 6 h incubation, the residual enzyme activity of rProROL in 20% ethanol and 10% ethanol were 81% and 88%, respectively. This tendency was found to be even better in the presence of methanol; the residual enzyme activity of rProROL in 20% methanol and 10% methanol were 91% and 97%, respectively. However, the lipase activity decreased dramatically when ethanol and methanol concentrations were increased up to 30%. Yang *et al.* reported that lipases from *Pseudomonas cepacia*, *Candida rugosa* and *Candida antarctica* were inactivated by 10% methanol in 30 min incubation [25]; therefore, rProROL seems to possess better methanol tolerance than these lipases.

#### 2.4.5. Substrate Specificity

**Figure 5.**
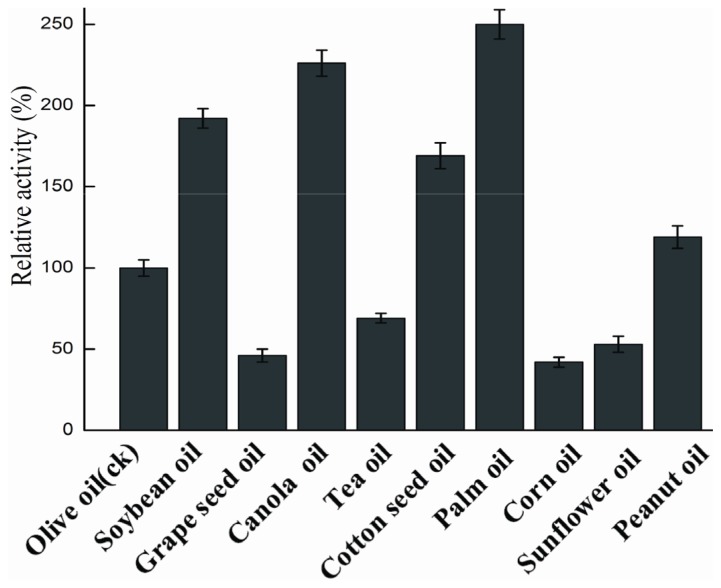
Substrate specificity of rProROL on various oils. Activities on each substrate are expressed as the percentage of that on olive oil. All measurements were carried out in triplicate.

The substrate preferences of rProROL were characterized with various triglycerides, fatty acid methyl esters and oil. As shown in Table 5 and Figure 5, relative activity on each substrate is expressed as the percentage of that on olive oil. For triglycerides, the lipase showed high activity towards saturated triglyceride-Tripalmitin (C16:0) and triglyceride-Trilaurin (C12:0). For fatty acid methyl ester, the lipase preferred long-chain fatty acid esters (C12, C16, C18). Similar substrate specificity towards triglycerides was reported for *Rhizopus delemar* lipase [26]. As shown in Figure 5, rProROL showed relative high activity on various emulsified oils (from 51% to 245%), especially on palm oil. The broad hydrolysis spectrum of various oil sources indicates that this recombinant lipase may play a significant part in the production of biodiesel [27].

## 3. Experimental Section

### 3.1. Strains, Plasmids and Materials

*Rhizopus oryzae* 3005 was purchased from the China Center of Industrial Culture Collection (Beijing, China). The *E. coli* strain Top 10 is routinely conserved in our laboratory. *Pichia pastoris* X-33, the expression vector pPICZαA and zeocin were purchased from Invitrogen (Carlsbad, CA, USA). Restriction enzymes, T4-DNA ligase and Pfu DNA polymerase were purchased from Sangon Biotech (Shanghai, China). The oligonucleotides were synthesized by the Shanghai Generay Company (Shanghai, China). The different lipase substrates were purchased from Sigma-Aldrich (St. Louis, MO, USA).

### 3.2. Vector Construction

The ProROL gene (GenBank Accession No. AF229435), was amplified by polymerase chain reaction methodology using genome DNA from *R. oryzae* as a template. The amplification primers containing the restriction sites for *EcoRI* and *XbaI* were designed as: PrF, 5′-CAGCGAATTCGTTCCTGTTTCTGGTAAATCTGG-3′ and PrR, 5′-GACGTCTAGATTACAAACAGCTTCCTTCGTT-3′. The PCR product were double digested with *EcoRI* (Sangon Biotech) and *XbaI* (Sangon Biotech), and then ligated into pPICZαA, forming pPICZαA-ProROL. Finally, the recombinant expressing vector pPICZαA-ProROL was used to transform *E. coli* Top 10. Through DNA sequencing, pPICZαA-ProROL was confirmed to contain the ProROL gene.

### 3.3. Transformation and Selection of *P. pastoris* Clones

*P. pastoris* X-33 was transformed with 10 μg of *Sac*I-linearized pPICZαA-ProROL vector by electro-transformation, according to Invitrogen’s recommendations. Transformants were plated on YPDS plates (10 g/L yeast extract, 20 g/L peptone, 20 g/L dextrose, 20 g/L agar, and 1 M sorbitol) containing 100 μg/mL Zeocin to isolate resistant clones. Then the Zeocin-resistant clones were shifted to YPD-rhodamine B-olive oil medium plates containing 0.08 g/L rhodamine B and 1% (*v*/*v*) emulsified olive oil. Transformed colonies were confirmed by both PCR and sequencing.

### 3.4. Shaking Flask Cultures

Thirty clones from the YPD-rhodamine B-olive oil medium plates were selected according to the size of the halos that formed around the colonies. The seeds were incubated in 10 mL of Buffered Glycerol-complex Medium (BMGY) [yeast extract 1% (*w*/*v*), peptone 2% (*w*/*v*), 100 mM potassium phosphate buffer at pH 6.0, yeast nitrogen base with no amino acids 1.34% (*w*/*v*), glycerol 1% (*w*/*v*), biotin 0.04% (*w*/*v*)] in a 100 mL shake flask and incubated at 30 °C and 200 rpm until the culture reached OD600 = 2.0–6.0. The cells were harvested by centrifugation and resuspended in 50 mL Buffered Methanol-complex Medium (BMMY) (containing 0.5% methanol instead of glycerol as the sole carbon source) and incubated at 30 °C and 200 rpm. The methanol induction temperature was set at 30 °C, and 0.7% (*v*/*v*) methanol was fed at 24-h intervals for 5 days. The activities of the lipase were checked at 24, 48, 72, 96, 120, 144 and 168 h. The colony with the highest activity was selected as the strain to ferment in the 50-L bioreactor.

### 3.5. High Cell Density Fermentation

The transformed strain showing the highest lipase activity in shake-flask culture was cultivated in high cell density fermentor. High cell density fermentation was carried out in 50-L bioreactor (Baoxing Co., Shanghai, China). Inoculum was cultured in BMGY medium. Cells were grown for 18–20 h a 30 °C on shaker of 200 rpm. Then, 10% (*v*/*v*) of the inoculum was inoculated into the 50-L bioreactor containing 20 L basal salt medium made of 0.47 g/L CaSO_4_·2H_2_O, 9.1 g/L K_2_SO_4_, 7.5 g/L MgSO_4_·7H_2_O, 6.2 g/L KOH, 13.35 mL/L H_3_PO_4_ (85%), 20.0 g/L glycerol and 1.5 mL Pichia trace metal 1 (PTM1) (Guangzhou Chemical Reagent Factory, Guangzhou, China). One liter PTM1 consists of 6 g CuSO_4_·5H_2_O, 0.08 g NaI, 3 g MnSO_4_·H_2_O, 0.5 g CoCl_2_, 20 g ZnCl_2_, 0.02 g H_3_BO_3_, 0.2 g Na_2_MoO_4_·2H_2_O, 65 g FeSO_4_·7H_2_O, 0.2 g biotin and 30 mL 6 N H_2_SO_4_. The temperature was controlled at 30 °C and the pH was maintained at 5.0 using NH_4_OH (28%) and H_3_PO_4_ (10%). The agitation rate was set at 500 rpm and the aeration rate was 40 L/min. When glycerol was used up, as indicated by an increased in dissolved oxygen (DO), 0.5% (*v*/*v*) methanol was added to induce expression the lipase. Feeding of methanol was linked to the dissolved oxygen (DO). When the initial methanol 0.5% (*v*/*v*) was depleted (indicated by an abrupt increase in DO), 80 g of 100% methanol solution containing of 1.2% (*v*/*v*) PTM1 was added automatically. The concentration of methanol was kept stable by monitoring the dissolved oxygen (OD) content and maintaining it at greater than 20%. The lipase activity of the supernatant and dry cell weight were monitored throughout the cultivation.

### 3.6. Biomass Analysis

Cell density was expressed as grams of dry cell weigh (DCW) per liter of broth, and was obtained by centrifuging 10 mL samples in a pre-weighted centrifuge tube at 8000 *g* for 10 min and washing twice with deionized water, then allowing the pellet to dry at 100 °C to constant weight.

### 3.7. Assay of Lipase Activity and Total Protein Concentration

The lipase activity in the culture filtrate was measured using olive oil as substrate. Ten percent (*v*/*v*) olive oil was emulsified in distilled water containing 2% (*w*/*v*) gum arabic as stabilizer using a homogenizer (Ultrtaturrax T25, Janke and Kunkel, IKA, Staufen, Germany) for 10 min at maximum speed. Twenty milliliters of substrate solution were heated to 40 °C and adjusted to pH 8.0. After addition of 5–20 μL of the enzyme solution, the activity was measured with a pH-stat (Metrohm, Herisau, Switzerland). Liberated fatty acids were titrated automatically with 0.01 N NaOH to maintain the pH constant at 8.0. One unit (U) of lipase activity was defined as the amount of enzyme that liberates 1 μmol fatty acid per minute under assay conditions. The protein content was determined according to the Bradford method using BSA as standard.

### 3.8. Purification of Lipase

After fermentation, cells from the cultures were removed by centrifuging at 10,000× *g* for 15 min. The supernatant was concentrated by ultrafiltration using a Millipore (Millipore Corporation, Billerica, MA, USA) set up according to the manufacturer’s instructions with a membrane of 10 kDa cut off, and subsequently dialyzed against 20 mM sodium phosphate buffer (PBS; pH 6.0) at 4 °C overnight. For further purification, the enzyme solution was loaded onto an SP Sepharose Fast Flow column (GE Healthcare, Uppsala, Sweden) (2.0 cm × 20 cm) equilibrated with 0.02 M PBS. Elution was performed with a linear gradient of 0–0.5 M NaCl at a rate of 2 mL/min. The active fractions were pooled, and the purity of lipase was analyzed by sodium dodecyl sulfate-polyacrylamide gel electrophoresis (Sangon Biotech).

### 3.9. Biochemical Characteristics of Recombinant Lipase

#### 3.9.1. Effect of pH and Temperature on Enzyme Activity and Stability

The activity of the lipase at different temperature and pH was measured by pH-stat assay using olive oil as the substrate. The effect of temperature on lipase stability was determined by incubating lipase solutions for 1 h in 50 mM Tris-HCl buffer (pH 8.0), at different temperatures. Residual enzyme activity was measured by pH-stat assy. For the pH stability, the enzyme was incubated at 30 °C in 50 mM acetic acid-sodium acetic acid buffer (pH 3.0–5.0), 50 mM Na_2_HPO_4_-NaH_2_PO_4_ buffer (pH 6.0–7.0), 50 mM Tris-HCl buffer (pH 8.0–9.0), NaHCO_3_ buffer (pH 10.0–11.0). After 24 h of incubation, the residual activity was determined as described previously.

#### 3.9.2. Effect of Metal Ions on Lipase Activity

The effect of metal ions on lipase activity was analyzed by incubating enzyme samples for 6 h at room temperature in 50 mM Tris-HCl buffer (pH 8.0), containing 1 mM of Ca^2+^, Mg^2+^, Na^+^, K^+^, Li^+^, Zn^2+^, Mn^2+^, Co^2+^, Hg^2+^, Ag^+^ and Fe^3+^. The residual activity was determined as described previously.

#### 3.9.3. Effect of Detergents on Lipase Activity

The effect of detergents on lipase activity was analyzed by incubating enzyme samples for 6 h at room temperature in 50 mM Tris-HCl buffer (pH 8.0), containing 1% (*v*/*v*) of the detergents (Triton X-100, Tween-80, Tween-20 and SDS) (Sangon Biotech). The residual activity was determined as described previously.

#### 3.9.4. Effect of Organic Solvents on Lipase

The effect of organic solvents on enzyme activity was determined by measuring residual activity after pre-incubation of 2 mL enzyme solution for 6 h at room temperature, in 2 mL of methanol, ethanol, acetone, isopropanol, glycerol, butanol, chloroform and hexane ether, respectively. Besides this, enzyme stability in various concentrations of methanol and ethanol were evaluated since enzyme stability in these solvents is highly desirable for the enzymatic biodiesel production.

#### 3.9.5. Substrate Specificity

For the determination of substrate specificity, 10% (*v*/*v*) triglycerides, including tributyrin, tricaprylin, trilaurin, tripalmitin, tristearin, olive oil and fatty acid methyl esters, including methyl butyrate, methyl caprylin, methyl laurate, methyl palmitate, methyl stearate were used as substrates with 2% (*w*/*v*) gum arabic as stabilizer in the pH-stat assay. Meanwhile, the activity of rProROL against a series of vegetable oils was determined in a similar manner.

## 4. Conclusions

In this study, ProROL was functionally expressed and secreted in *P. pastoris*. To our knowledge, this is the first report of heterologous high-level expression and characterization of ProROL in *P. pastoris*. The maximum lipase activity of rProROL was 21,000 U/mL, which was obtained in a fed-batch cultivation in 50-L bioreactor. Compared with the expression of ProROL in *E. coli*, the expression of ProROL in *P. pastoris* has many advantages, such as high levels of productivity (in *P. pastoris*, the activity of rProROL could reach 21,000 U/mL, while in *E. coli*, the activity of rProROL is only about 166 U/mL), the rProROL is in the supernatant, and cell disruption is not necessary. Compared with the expression of ProROL in *S. cerevisiae*, the expression of ProROL in *P. pastoris* has high levels of productivity (in *S. cerevisiae* the activity of rProROL could reach only 2.88 U/mL, after 120 h of cultivation in 200 mL YPD medium), and the *P. pastoris* was found to be more suitable for high-density fermentation than *S. cerevisiae*. The optimum pH and temperature of the rProROL were pH 9.0 and 40 °C, respectively. The lipase was stable from pH 4.0 to 9.0 and from 25 to 55 °C. Metal ion Ca^2+^ increased the activity of rProROL by 11%. The activity of lipase increased slightly in the presence of Mg^2+^, K^+^, Na^+^ and Triton X-100. Tween-80, Tween-20 and SDS decreased lipase activity. The lipase showed high activity toward long-chain fatty acid methyl esters (C12–C16).

## Supplementary Information



## Figures and Tables

**Table 1.** Purification of rProROL from 100 mL culture supernatant.

Purification steps	Total activity (U)	Total protein (mg)	Specific activity (U/mg)	Yield (%)	Purification factor
Supernatant	2,100,000	950	2,210	100	1
Ultrafiltration	1,974,000	740	2,667	94	1.20
SP-Sepharose FF	1,512,000	226	6,690	72	3.02

**Table 2.** Influence of various metal ions (1 mM) on the activity of rProROL. The effect of metal ions on lipase activity was analyzed by incubating enzyme samples for 6 h at room temperature in 50 mM Tris-HCl buffer (pH 8.0), containing 1 mM of metal ions. The activity was measured with a pH-stat (Metrohm, Herisau, Switzerland) and using olive oil as substrate at pH 9.0, 40 °C. The activity of rProROL was determined in the buffer with no addition of metal ions and set as 100%. All measurements were carried out in triplicate.

Metal ions (1 mM)	Relative activity (%)	Metal ions (1 mM)	Relative activity (%)
Control	100	Control	100
Ca^2+^	111 ± 2	Co^2+^	84 ± 3
Zn^2+^	42 ± 1	K^+^	106 ± 3
Li^+^	89 ± 3	Na^+^	105 ± 4
Fe^3+^	76 ± 3	Hg^+^	0
Mn^2+^	88 ± 4	Ag^+^	5 ± 1
Mg^2+^	107 ± 3	EDTA	90 ± 5

**Table 3.** Influence of various organic solvents on the activity of rProROL. Enzyme samples were mixed with solvents (50%, *v*/*v*) and incubated for 6 h in a rotary shaker set at 160 rpm and 25 °C, prior to determining the residual activity. The activity was measured with a pH-stat (Metrohm) and using olive oil as substrate at pH 9.0, 40 °C. All measurements were carried out in triplicate.

Organic solvents	Relative activity (%)	Organic solvents	Relative activity (%)
Control	100	Control	100
Methanol	22 ± 3	Acetone	27 ± 3
Ethanol	14 ± 1	Butanol	11 ± 2
Isopropanol	17 ± 1	Chloroform	16 ± 2
Hexane	61 ± 4	Glycerol	104 ± 6

**Table 4.** Influence of methanol and ethanol concentrations on the activity of rProROL. Enzyme samples were incubated with various concentrations of methanol and ethanol for 6 h in a shaker set at 160 rpm and 25 °C. The activity was measured with a pH-stat (Metrohm) and using olive oil as substrate at pH 9.0, 40 °C. All measurements were carried out in triplicate.

Solvent concentrations (%)	Relative activity (%)	

	Methanol	Ethanol
0	100	100
5	98 ± 5	101 ± 3
10	97 ± 4	88 ± 4
20	91 ± 3	81 ± 4
30	61 ± 4	41 ± 3
40	22 ± 1	16 ± 2

**Table 5.** Substrate specificity of rProROL on various triglycerides and fatty acid methyl esters. Activities on each substrate are expressed as the percentage of that on olive oil. All measurements were carried out in triplicate.

Substrates	Relative activity (%)	Substrates	Relative activity (%)
Olive oil	100	Olive oil	100
Tributyrin (C4)	23 ± 2	Methyl butyrate (C4)	5 ± 2
Tricaprylin (C8)	134 ± 6	Methyl caprylin (C8)	8 ± 2
Trilaurin (C12)	156 ± 4	Methyl laurate (C12)	9 ± 1
Tripalmitin (C16)	178 ± 4	Methyl palmitate (C16)	11 ± 2
Tristearin (C18)	45 ± 3	Methyl stearate (C18)	10 ± 2
