# Supplementary material for: High-Level Expression of Pro-Form Lipase from Rhizopus oryzae in Pichia pastoris and Its Purification and Characterization
Source: Int J Mol Sci. 2013 Dec 24;15(1):203–17. doi: 10.3390/ijms15010203 (PMC3907806; doi:10.3390/ijms15010203)
Supplement: Supplementary file 1 [file ijms-15-00203-s001.pdf]

Supplementary Information

Shaking Flask Cultures

One hundred randomly picked zeocin resistant positive clones on the solid selective medium (YPDS containing zeocin) were transferred on YPD-rhodamine B-olive oil medium plates to check lipase expression level. Lipase expression was induced by adding 0.2 mL of methanol onto the lid covering the plates. 10 colonies that had larger halos were selected and cultivated in shaking flask. As shown in Figure S1, the R2 had the activity of 450 U/mL induced by methanol after 144 h and the R1 had the highest activity of 780 U/mL after 144 h cultivation which was about 1.73 time than R1.

Figure S1. Lipase activity of X33/pPICZαA-ProROL in Buffered Methanol-complex Medium (BMMY) medium at 30 °C and 250 r/min induced by methanol. The lipase activity was measured with a pH-stat (Metrohm) and using olive oil as substrate at pH 9.0, 40 °C.

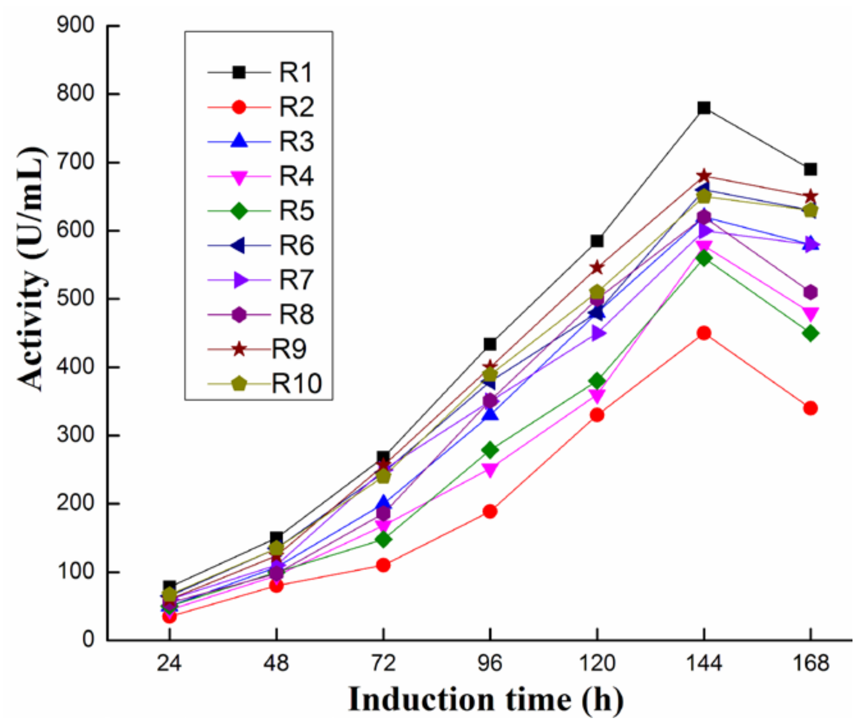

© 2013 by the authors; licensee MDPI, Basel, Switzerland. This article is an open access article distributed under the terms and conditions of the Creative Commons Attribution license (http://creativecommons.org/licenses/by/3.0/).
